# Metabolomics-based search for lung cancer markers among patients with different smoking status

**DOI:** 10.1038/s41598-024-65835-2

**Published:** 2024-07-04

**Authors:** Agnieszka Klupczynska-Gabryszak, Evangelia Daskalaki, Craig E. Wheelock, Mariusz Kasprzyk, Wojciech Dyszkiewicz, Marcin Grabicki, Beata Brajer-Luftmann, Magdalena Pawlak, Zenon J. Kokot, Jan Matysiak

**Affiliations:** 1 Department of Inorganic and Analytical Chemistry, Poznan University of Medical Sciences, Poznan, Poland; 2 Unit of Integrative Metabolomics, Institute of Environmental Medicine, Karolinska Institutet, Stockholm, Sweden; 3 Department of Respiratory Medicine and Allergy, Karolinska University Hospital, Stockholm, Sweden; 4 Department of Thoracic Surgery, Poznan University of Medical Sciences, Poznan, Poland; 5 Department of Pulmonology, Allergology and Respiratory Oncology, Poznan University of Medical Sciences, Poznan, Poland; 6 Faculty of Health Sciences, Calisia University, Kalisz, Poland

**Keywords:** Non-small cell lung cancer, Metabolic profile, Tobacco smoking, Biomarker, Mass spectrometry, Lung cancer, Diagnostic markers, Translational research, Mass spectrometry

## Abstract

Tobacco smoking is the main etiological factor of lung cancer (LC), which can also cause metabolome disruption. This study aimed to investigate whether the observed metabolic shift in LC patients was also associated with their smoking status. Untargeted metabolomics profiling was applied for the initial screening of changes in serum metabolic profile between LC and chronic obstructive pulmonary disease (COPD) patients, selected as a non-cancer group. Differences in metabolite profiles between current and former smokers were also tested. Then, targeted metabolomics methods were applied to verify and validate the proposed LC biomarkers. For untargeted metabolomics, a single extraction-dual separation workflow was applied. The samples were analyzed using a liquid chromatograph-high resolution quadrupole time-of-flight mass spectrometer. Next, the selected metabolites were quantified using liquid chromatography-triple-quadrupole mass spectrometry. The acquired data confirmed that patients’ stratification based on smoking status impacted the discriminating ability of the identified LC marker candidates. Analyzing a validation set of samples enabled us to determine if the putative LC markers were truly robust. It demonstrated significant differences in the case of four metabolites: allantoin, glutamic acid, succinic acid, and sphingosine-1-phosphate. Our research showed that studying the influence of strong environmental factors, such as tobacco smoking, should be considered in cancer marker research since it reduces the risk of false positives and improves understanding of the metabolite shifts in cancer patients.

## Introduction

Tobacco smoking is the main etiological factor of all histological types of lung cancer (LC), accounting for 80 to 90% of lung cancer cases in the Western world^1,2^. The risk of lung cancer among smokers is about 20–50-fold higher than that among never-smokers^3^. The carcinogenic effect of cigarette smoking on the lung was recognized in the 1950s based on research conducted in the fields of epidemiology, cellular pathology, chemical analysis, and animal studies^4^. Tobacco and tobacco smoke contain more than 9500 identified chemicals. According to a recently published update, a total of 83 tobacco and tobacco smoke components were classified as carcinogens by the International Agency for Research on Cancer in 2022^5^. Polycyclic aromatic hydrocarbons, nicotine-derived nitrosamine ketones, volatiles, such as 1,3-butadiene, and metals, such as cadmium, are known as major lung carcinogens^6^. The best form of lung cancer prevention (primary, secondary and tertiary) is to stop smoking and limit environmental exposure to tobacco smoke^7^. Many people make such an effort, which is why patients with newly diagnosed lung cancer include active smokers and people who have given up their tobacco addiction^8^.

Metabolomics-based technologies analyze a large number of endogenous and exogenous low-molecular-weight compounds simultaneously, providing information about the metabolic status of living organisms^9^. Levels of these compounds are influenced by such factors as genes, diseases, lifestyle, and environment. Therefore, the metabolome reflects both endogenous processes and exogenous exposures, making metabolomics an especially suitable tool for studying multifactorial diseases. To highlight the importance of the environment in cancer etiology, the concept of the exposome was proposed^10^. A study of exposome focuses on non-genetic contributions to individual diseases by measuring the cumulative effects of environmental exposure and associated biological responses. Exposomics opens new opportunities in epidemiology research, especially of lung diseases^11–14^.

The metabolomics approach has been used for the identification of markers of tobacco smoke exposure and better characterization of smoking-related disease risks. Targeted metabolomics studies revealed 23 lipid metabolites as nicotine-dependent biomarkers suggesting damage of cell membranes by cigarette smoke^15^. Moreover, a follow-up study demonstrated that smoking-related alterations in metabolite levels are reversible after smoking cessation^16^, which is particularly interesting as a great percentage of lung cancer patients are former smokers. The impact of cigarette smoking on the metabolome was also investigated using untargeted strategies. Apart from nicotine metabolites, metabolites involved in vitamin, amino acid, benzoate, steroid, and carbohydrate pathways were identified as smoking-associated^17,18^. An altered fatty acid profile, with increased monounsaturated fatty acids, and glycerophospholipid profile was also indicated in the blood of smokers^19,20^.

LC has been the leading cause of cancer death worldwide in both sexes for years^21^. Often, LC is diagnosed when advanced-stage disease is present^22^. Due to the high morbidity and mortality of LC, recent years have abounded in metabolomics studies of LC^23,24^. However, the impact of tobacco smoking on the metabolic profiles of patients has been neglected in LC biomarker studies. A lack of matching in terms of cigarette smoking between the LC group and non-cancer group was observed^25–28^, whereas some studies presented no data on the smoking history of patients^29–31^. Although findings of metabolomics investigations in LC are encouraging, little is known whether the observed differences in the metabolome result from the development of cancer or if they are associated with chronic smoking. Smoking exposure is found in 85–90% of patients diagnosed with either LC or chronic obstructive pulmonary disease (COPD)^32^. Therefore, the selection of controls among patients with COPD is one of the strategies for obtaining a balanced group in terms of smoking habits that can be compared with LC patients^25,33,34^. In the review on LC metabolomics research prepared by Yu et al.^23^, only one out of 32 studies included in the review employed the COPD group as a comparative group for the LC group. Similarly, in the review on LC metabolic biomarkers written by Xu et al.^35^, one out of 22 cited articles included the COPD group as a comparative group for the LC group.

The study aimed to perform serum metabolomics analysis of newly diagnosed LC patients and patients with COPD, who were selected as a control, non-cancer group. A novelty of the study is patient categorization according to their smoking status, which allowed us to distinguish molecular markers associated with LC from smoking-related alterations in the metabolic profile. We applied a multi-step biomarker selection workflow. First, a high-resolution quadrupole time-of-flight (Q-TOF) mass spectrometry-based metabolomics was used for untargeted profiling of a broad spectrum of metabolites in the collected sera. Then, the putative LC markers were verified using targeted triple-quadrupole mass spectrometry-based methodologies and next validated using a new set of samples.

## Results

### Characteristics of the individuals included in the study

A total of 155 patients were included in our study. Patients were divided based on their smoking status and belonged to either the discovery set or the validation set (Table 1). Each LC subgroup had the highest percentage of patients in stage I cancer, which allowed us to study changes in metabolic profiles occurring in the early stage of the disease. Forty-four percent of LC patients had adenocarcinoma, whereas fifty-three percent had squamous cell carcinoma. The COPD group was matched to the LC group and consisted of individuals of the same ethnic origin (Caucasians). No significant difference in age and BMI between the LC group and the COPD group was observed in both the discovery set and validation set, as calculated by the statistical tests (*p* < 0.05). Moreover, the majority of each group and subgroup of patients were men (Table 1).

**Table 1:** Characteristics of study participants: newly diagnosed lung cancer (LC) patients and patients with chronic obstructive pulmonary disease (COPD).

		Discovery and verification set (n = 100)				Validation set (n = 55)			
		LC patients (n = 50)		COPD patients (n = 50)		LC patients (n = 28)		COPD patients (n = 27)	
		Current smokers	Former smokers	Current smokers	Former smokers	Current smokers	Former smokers	Current smokers	Former smokers
N		25	25	25	25	13	15	10	17
Age, years	Mean ± SD	63 ± 6.4	67 ± 5.2	61 ± 5.8	69 ± 8.3	64 ± 6.6	66 ± 3.4	63 ± 8.2	67.8 ± 10.4
	Median	62	68	61	70	65	65	63	66
Sex, n	Female	7	8	8	7	3	6	4	2
	Male	18	17	17	18	10	9	6	15
BMI, kg/m^2^	Mean ± SD	25.2 ± 4.5	26.9 ± 3.9	28.2 ± 6.8	26.4 ± 4.9	24.9 ± 4.0	26.0 ± 5.1	24.7 ± 9.1	30.4 ± 6.2
	Median	25.6	27.5	27.3	26.3	25.4	25.2	22.1	32.3
Smoking intensity, packyears	Mean ± SD	41.7 ± 13.0	29.3 ± 18.9	39.5 ± 13.4	30.3 ± 13.5	38.1 ± 15.5	24.9 ± 8.7	42.8 ± 20.4	46.2 ± 16.1
	Median	40	27	40	32	40	20	43	48
Smoking cessation time, years	2–5 years	n/a	9	n/a	6	n/a	5	n/a	4
	6–10 years	n/a	5	n/a	6	n/a	1	n/a	3
	11–15 years	n/a	8	n/a	6	n/a	5	n/a	5
	> 15 years	n/a	3	n/a	7	n/a	4	n/a	5
Clinical stage according to TNM classification, 7th ed, n	I	13	11	n/a	n/a	6	6	n/a	n/a
	II	8	6	n/a	n/a	4	5	n/a	n/a
	III	4	8	n/a	n/a	1	4	n/a	n/a
	Unspecified	–	–	n/a	n/a	2	–	n/a	n/a
Histology, n	Adenocarcinoma	11	10	n/a	n/a	5	8	n/a	n/a
	Squamous-cell carcinoma	12	15	n/a	n/a	7	7	n/a	n/a
	Other or unspecified	2	–	n/a	n/a	1	–	n/a	n/a

### Untargeted metabolomics analysis: discovery stage

Based on the acquired LC-HRMS profiles, 124 distinct metabolites were identified: 66 in HILIC separation mode and 58 in RP separation mode. Some metabolites were detected in both positive and negative ionization modes. The selection of polarity for a particular compound was based on signal intensity and %CV calculation of the QC samples. Due to peak saturation, presence in a blank sample, or high %CV value (above 15%) calculated based on QC samples a few metabolites were removed from the downstream statistical analysis. Moreover, theophylline and its metabolites (methyluric acid and dimethyluric acid) were also excluded from further comparisons of metabolic profiles between patients. Theophylline is a bronchodilator used in the treatment of COPD and the majority of patients with COPD included in the study were taking theophylline. Therefore, significantly higher levels of theophylline and its metabolites were observed in their sera (data not shown). This difference did not result from cancer status or smoking status. It showed that in cancer metabolomics, information on taken drugs should be included in data interpretation. To sum up, 103 metabolites were retained for further statistical analysis (Supplementary Table S5).

To ensure the quality of data acquired and to monitor the LC–MS system performance, QC samples were used. PCA showed tight clustering of the QC samples in both separation modes—HILIC and RP-LC (Supplementary Fig. S1A,C). This indicated that the instrument drift was minimal and confirmed high stability and repeatability of the system. The non-supervised multivariate PCA was also applied to investigate separation trends among samples collected from LC and COPD patients. However, PCA analysis revealed no clear separation between the studied groups and subgroups: LC current smokers, LC former smokers, COPD current smokers, and COPD former smokers (Supplementary Fig. S1).

To further assess cancer-related and smoking-related alterations in the metabolic profiles of the patients, volcano plots were constructed. The volcano plot exhibited the relationship between the FDR-corrected *P* values of a statistical test and a fold change. This univariate analysis of LC-HRMS data was performed for each dataset separately: the HILIC dataset (Table 2) and RP-LC dataset (Table 3) and included the division of the patients into subgroups not only depending on the LC occurrence but also depending on their smoking habit.

**Table 2:** Results of volcano plot analysis for HILIC dataset. Important metabolic features (marker candidates) occurring in all types of comparisons between lung cancer (LC) patients and chronic obstructive pulmonary disease (COPD) patients were bolded. Directions of comparison in fold change analysis: LC/COPD when grouping variable was cancer status; current smokers/former smokers when grouping variable was smoking status.

The grouping variable—cancer status								
A. LC vs COPD all patients			B. LC vs COPD current smokers			C. LC vs COPD former smokers		
Metabolite	Fold change	FDR-adjusted *P* value	Metabolite	Fold change	FDR-adjusted *P* value	Metabolite	Fold change	FDR-adjusted *P* value
**Glutamic acid**	**0.70**	** < 0.0001**	**Allantoin**	**0.64**	** < 0.0001**	Aspartic acid	0.69	< 0.0001
**Allantoin**	**0.60**	** < 0.0001**	**Glutamic acid**	**0.70**	** < 0.0001**	**Glutamic acid**	**0.70**	** < 0.0001**
**Succinic acid**	**0.71**	** < 0.0001**	**Succinic acid**	**0.70**	**0.0007**	**Inosine**	**2.50**	** < 0.0001**
Aspartic acid	0.75	< 0.0001	Catechol	0.54	0.0069	**Allantoin**	**0.57**	** < 0.0001**
**Inosine**	**2.65**	** < 0.0001**	Malic acid	0.74	0.0091	**Succinic acid**	**0.71**	** < 0.0001**
Hypotaurine	0.66	< 0.0001	**Inosine**	**2.83**	**0.0213**	Hypotaurine	0.57	0.0001
Malic acid	0.73	< 0.0001				Guanosine	2.28	0.0015
Guanosine	2.23	< 0.0001				Malic acid	0.72	0.0027
Fucose	0.54	0.0070				Tyrosine	0.75	0.0066

The grouping variable—smoking status								
D. current smokers vs former smokers			E. LC current smokers vs LC former smokers			F. COPD smokers vs COPD former smokers		
Metabolite	Fold change	FDR-adjusted *P* value	Metabolite	Fold change	FDR-adjusted *P* value	Metabolite	Fold change	FDR-adjusted *P* value
Malic acid	0.76	0.0040	Aconitic acid	0.77	0.0136	Catechol	1.76	0.0201
						Malic acid	0.75	0.0201

**Table 3:** Results of volcano plot analysis for RP-LC dataset. Important metabolic features (marker candidates) occurring in all types of comparisons between lung cancer (LC) patients and chronic obstructive pulmonary disease (COPD) patients were bolded. Directions of comparison in fold change analysis: LC/COPD when grouping variable was cancer status; current smokers/former smokers when grouping variable was smoking status.

The grouping variable—cancer status								
A. LC vs COPD all patients			B. LC vs COPD current smokers			C. LC vs COPD former smokers		
Metabolite	Fold change	FDR-adjusted *P* value	Metabolite	Fold change	FDR-adjusted *P* value	Metabolite	Fold change	FDR-adjusted *P* value
**Sphingosine-1-phosphate (d18:1)**	**0.69**	** < 0.0001**	**Sphingosine-1-phosphate (d18:1)**	**0.68**	**0.0002**	**1,2-Dioleoylglycerol**	**1.42**	**0.0149**
**1,2-Dioleoylglycerol**	**1.44**	** < 0.0001**	Lyso-PAF C-16	0.69	0.0004	**Sphingosine-1-phosphate (d18:1)**	**0.71**	**0.0149**
Lyso-PAF C-16	0.72	< 0.0001	Arachidonic acid	0.70	0.0018			
2-O-Ethyl PAF C-16	0.75	< 0.0001	2-O-Ethyl PAF C-16	0.73	0.0030			
Sphinganine-phosphate (d18:0)	0.72	0.0003	Stearoylethanolamide	0.74	0.0055			
1-Linoleoylglycerol	1.97	0.0006	Sphinganine-phosphate (d18:0)	0.72	0.0063			
1-Arachidonoylglycerol	2.00	0.0022	1-Linoleoylglycerol	0.32	0.0086			
Chenodeoxycholic acid	0.31	0.0030	**1,2-Dioleoylglycerol**	**0.68**	**0.0094**			
Arachidonic acid	0.76	0.0037	1-Arachidonoylglycerol	0.31	0.0215			
Eicosapentaenoic acid	0.67	0.0125	Eicosapentaenoic acid	0.62	0.0306			
			Ceramide (d18:1/14:0)	0.74	0.0359			

The grouping variable—smoking status								
D. current smokers vs former smokers			E. LC current smokers vs LC former smokers			F. COPD current smokers vs COPD former smokers		
Metabolite	Fold change	FDR-adjusted *P* value	Metabolite	Fold change	FDR-adjusted *P* value	Metabolite	Fold change	FDR-adjusted *P* value
No records found	–	–	Adrenic acid	0.72	0.0436	No records found	–	–

In the volcano plot comparing HILIC-based metabolic profiles of LC patients and COPD patients, the following nine variables met the set criteria: glutamic acid, allantoin, succinic acid, aspartic acid, inosine, hypotaurine, malic acid, guanosine, and fucose (Table 2A). However, patient stratification by smoking status revealed different sets of cancer marker candidates. Volcano plot analysis performed using samples collected from LC smokers and COPD smokers demonstrated six metabolites as putative LC markers (Table 2B), which did not fully overlap with the results in the previous comparison. In turn, the comparison of metabolic profiles of ex-smokers showed nine LC marker candidates (Table 2C). The other three volcano plots were constructed to examine the impact of smoking status on the metabolic profiles of patients. The performed analyses showed that lowered malic acid is an important feature of the serum of smokers (Table 2D). It should be noted that the same metabolite was among the proposed cancer markers. Therefore, the observed variations in serum level of malic acid are associated with both cancer and smoking habits, which excludes it as a marker candidate for LC. In addition, aconitic acid and catechol were also found as metabolites differentiating subgroups of current smokers and former smokers (Table 2E,F).

An analogous series of statistical tests was conducted for the RP-LC-based metabolic profiles. In RP-LC dataset 10 compounds were identified as important features differentiating LC patients from COPD patients (Table 3A). However, only 2 metabolites were common among all types of comparisons between LC and COPD patients (Table 3A–C).

To sum up, an additional patient separation on subgroups based on the smoking status exhibited different LC marker candidates within each type of comparison. The performed tests allowed for the selection of LC candidates that were subsequently subjected to a verification stage (absolute quantification) and then a validation stage (external validation using a new set of samples). We selected only metabolites that met two criteria: they were identified as important features in all types of comparisons between LC patients and COPD patients and their levels were unrelated to the smoking status of the patients (they did not emerge as differential metabolites in comparisons between current smokers and past smokers). From the HILIC dataset analysis, four metabolites were chosen for targeted quantification: glutamic acid, allantoin, succinic acid, and inosine. From the RP dataset analysis, two metabolites were chosen for targeted quantification: sphingosine-1-phosphate (d18:1) and 1,2-dioleoylglycerol.

### Targeted metabolomics analysis: verification and validation stage

After the selection of six putative LC markers via untargeted metabolomics analysis, we developed targeted methods to perform absolute quantification of these metabolites in the collected patient sera. The targeted triple quadrupole MS-based methods were validated according to EMA guidelines^36^. All validation steps confirmed their reliability and appropriateness for the metabolite determination in human serum (Supplementary Table S2).

**Figure 1:**
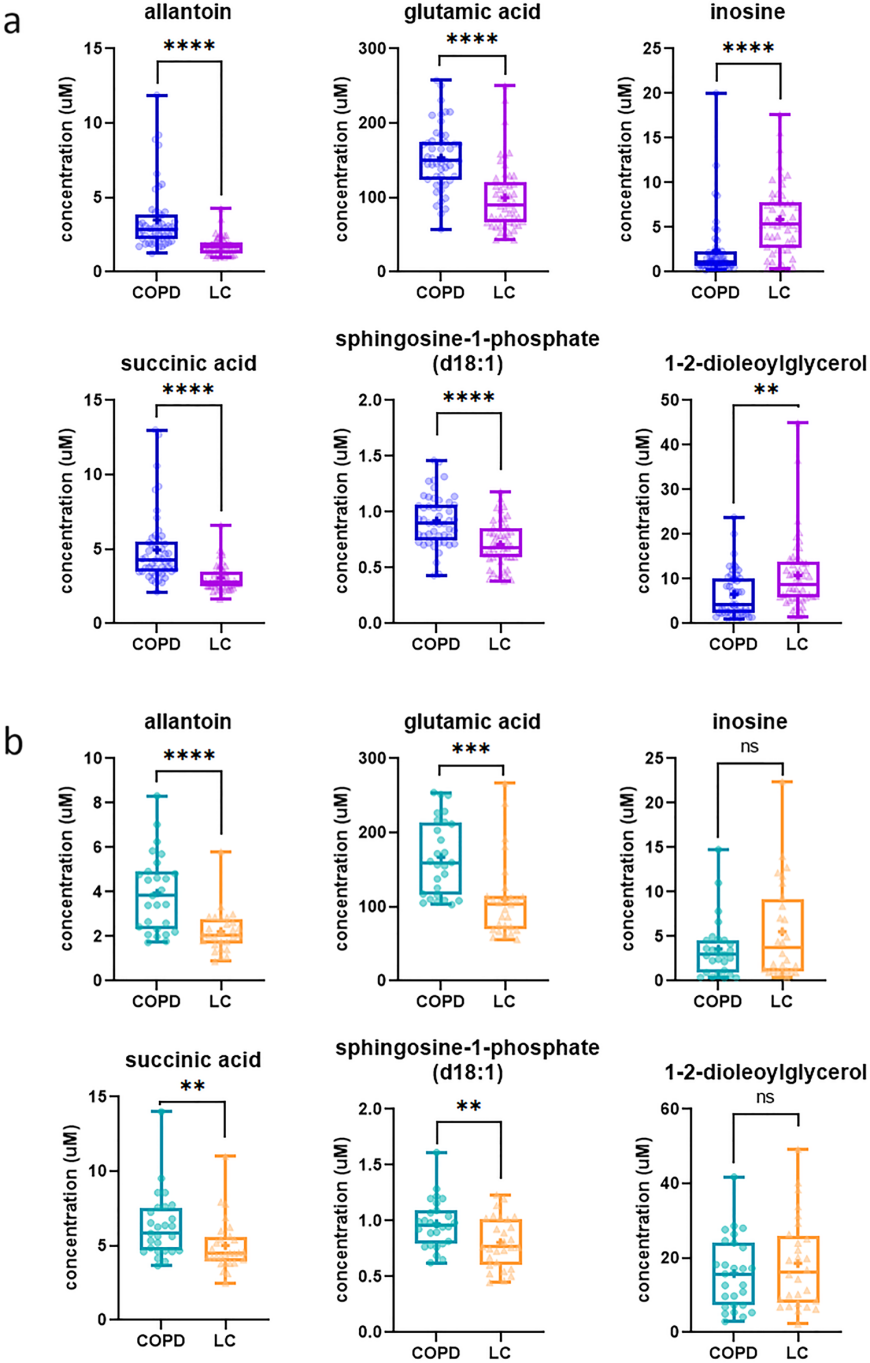
Box and whiskers plots showing concentration distributions of the selected metabolites obtained from targeted quantitative analysis of the subjects from the discovery set (**a**) and the validation set (**b**). The line in the middle of the box represents the median, whereas “+” indicates the mean. *LC* lung cancer, *COPD* chronic obstructive pulmonary disease. ****P ≤ 0.0001; ***P ≤ 0.001; **P ≤ 0.01, *ns* not significant.

First, the targeted methods were used to measure the metabolite concentrations in the discovery set of patients (to perform a quantitative verification step). The conducted targeted metabolomics analysis confirmed significant differences in the concentration of all identified putative metabolites between the analyzed groups of patients (Fig. 1a). The serum levels of allantoin, glutamic acid, succinic acid, and sphingosine-1-phosphate (d18:1) were significantly lowered in the LC group compared with the COPD group, whereas the serum levels of inosine and 1–2-dioleoylglycerol were significantly elevated in the LC group. The determined concentrations of the putative LC markers are shown in Table 4.

**Table 4:** Concentration levels of the six lung cancer candidate markers determined using targeted metabolomics analysis in the discovery set and the validation set of samples. *LC* lung cancer, *COPD* chronic obstructive pulmonary disease.

Metabolite	Verification step					Validation step				
	LC group (n = 50)		COPD group (n = 50)		*P* value	LC group (n = 28)		COPD group (n = 27)		*P* value
	Mean (μM)	Median (μM)	Mean (μM)	Median (μM)		Mean (μM)	Median (μM)	Mean (μM)	Median (μM)	
Allantoin	1.77	1.71	3.49	2.88	< 0.0001	2.22	2.07	3.95	3.84	< 0.0001
Glutamic acid	99.97	89.55	153.4	149.5	< 0.0001	109.9	103.0	166.7	159.5	0.0001
Inosine	5.88	5.38	2.36	1.15	< 0.0001	5.54	3.72	3.60	2.96	0.2665
Succinic acid	3.07	2.79	4.94	4.27	< 0.0001	5.00	4.49	6.31	5.83	0.0033
Sphingosine-1-Phosphate (d18:1)	0.71	0.68	0.92	0.90	< 0.0001	0.81	0.77	0.97	0.96	0.0074
1–2-Dioleoylglycerol	10.79	8.81	6.57	4.33	0.0011	18.50	16.19	15.83	15.74	0.4953

Next, the targeted methodologies were applied for the analysis of a new set of samples to conduct an external validation. The analysis of a validation set of samples allowed us to better assess the differentiating potential of the previously chosen putative LC markers. The performed experiments showed the same direction of change in the metabolite concentrations in the LC group compared with the COPD group. However, the differences were statistically significant in the case of four out of six metabolites that were selected as LC marker candidates, namely: allantoin, glutamic acid, succinic acid, sphingosine-1-phosphate (d18:1) (Fig. 1).

To assess the robustness of the six putative LC markers identified by the untargeted metabolomics, we also compared their levels between groups of LC and COPD patients separated additionally based on their smoking status. In the verification step, we observed statistically significant differences in the concentrations of all six marker candidates in the comparison of LC current smokers vs COPD current smokers as well as in the comparison of LC former smokers vs COPD former smokers (Supplementary Tables S6 and S7). In the validation step, patient stratification by smoking status demonstrated statistically significant differences only in the levels of allantoin, glutamic acid, and succinic acid (Supplementary Table S6). The downregulation of sphingosine-1-phosphate (d18:1) in LC patients was confirmed in the validation set of samples in the subgroups of current smokers as well as former smokers; however, without statistical significance (the obtained *P* values were close to 0.05). The upregulation of inosine in LC patients was confirmed in the comparison of LC current smokers vs COPD current smokers, whereas the upregulation of 1–2-dioleoylglycerol in LC patients was confirmed in comparison of LC former smokers vs COPD former smokers (Supplementary Tables S6 and S7).

## Discussion

Tobacco smoking is the major cause of LC that might affect the patients’ metabolome. Therefore, the study aimed to investigate whether the observed metabolic shift in lung cancer patients was also associated with the smoking status of patients. To address this, we applied a multi-step biomarker selection along with an external validation. First, untargeted metabolomics profiling was applied for the initial screening of changes in serum metabolic profile occurring between LC and COPD patients as well as current smokers and former smokers. Then, targeted metabolomics methods with higher accuracy and repeatability were applied to verify and validate the proposed LC biomarkers. Cancer metabolomics studies frequently take advantage of the possibilities given by untargeted strategy^37^. It gives the opportunity to look at a wide metabolic profile of a sample covering compounds from different metabolite classes; however, it does not provide quantitative data^38^. The targeted strategy quantifies metabolites selected a priori, requires metabolite standards, and has higher reproducibility^9^. Therefore, it can verify and validate metabolome changes discovered via an untargeted approach. Unfortunately, numerous LC metabolomics studies lacked the targeted step^39^. They reported the results of only initial metabolic screening and ended up showing differences in signal intensities without subsequently converting the observed differences into concentration units. Untargeted and targeted approaches provide complementary information and should not be used interchangeably due to their merits and limitations^9^. The workflow presented in the current study, incorporating both approaches, should be more prevalent and the targeted methods should be an important part of any metabolomics study.

One of the key and most challenging aspect of comparative omics studies, aiming to search for cancer markers, is the selection of a control group (a comparative group). In previous LC metabolomics investigations healthy subjects were utilized as a control group, whereas others used COPD patients as a control group^25,33,34^. Another solution is employing more than one control group, including COPD patients or individuals with other pulmonary diseases^40^. LC and COPD may share similar pathogenic mechanisms—inflammation and oxidative stress^41^. Thus, the distinction between these two diseases should be addressed in metabolomics studies. The previously conducted research comparing metabolic fingerprints of LC patients and COPD patients indicated that carcinogenesis had a stronger impact on human metabolome composition than COPD^25^.

The performed untargeted metabolomics profiling allowed us to look at patients' metabolic profiles from a broader perspective. We hypothesized that LC patients’ categorization according to their smoking status could lead to the identification of different alterations in metabolite profiles. As expected, tobacco smoking influenced the metabolite profiles of the patients. Among metabolites, whose levels differed between current smokers and former smokers were malic acid, catechol, and adrenic acid. Catechol occurs in natural sources, such as fruits and vegetables, but is also present in cigarette smoke^42^. Our study demonstrated elevated level of this metabolite in current smokers with COPD compared with former smokers with COPD as well as current smokers with COPD compared with current smokers with LC (Table 2). The performed statistical analysis also showed that cancer-related alterations in serum metabolome are more visible than smoking-related alterations, which is manifested in the number of discriminating variables in specific comparisons (Tables 2, 3).

The untargeted metabolomics experiments showed that metabolite profiles are influenced by the smoking status and xenobiotics taken by patients (theophylline). Therefore, each metabolomics report should contain a detailed patient characteristic covering not only demographic data but a vast clinical characteristic including comorbidities, drug intake, lifestyle factors, and diet. In other metabolomics studies, synthetic molecules, such as bisphenol A, were identified as LC metabolite markers^43^, which raises many questions about their biological significance and clinical utility. Identification of theophylline as an altered metabolite in LC in the current study showed that the interpretation of metabolomics data should be done with caution.

On the basis of the performed untargeted metabolomics profiling, we proposed six putative LC markers that distinguish LC patients from COPD patients: allantoin, glutamic acid, inosine, succinic acid, sphingosine-1-phosphate (d18:1), and 1–2-dioleoylglycerol. To be clinically useful, the metabolomics analysis should provide absolute metabolite quantitation. Therefore, the results obtained in untargeted metabolite profiling were then verified to obtain data on the absolute concentrations of the selected compounds. Of particular interest is the high level of agreement observed between results obtained in untargeted profiling and targeted verification.

In the current study, we sought to compare metabolite shifts between current smokers and former smokers within the LC patient group. The previous targeted metabolomics study demonstrated that smoking-related alterations in serum metabolite levels are reversible after smoking cessation^16^. Therefore, we applied strict criteria to include an LC patient as a current smoker or a past smoker. However, in clinical practice, a substantial group of LC patients are people who quit smoking shortly before cancer diagnosis^8^. Among LC patients excluded from the current study were many patients who had stopped smoking 2 months before biopsy or even later. Therefore, they constituted a borderline group between current smokers and former smokers. Thus, one of our aims was to identify the changes in patient metabolome that are present in all newly diagnosed LC patients, both smokers and former smokers. Separate statistical analyses were performed for current smoker subgroups and former smoker subgroups to determine whether the putative LC markers maintain their discriminating potential for LC patients with different smoking statuses (Supplementary Tables S6 and S7).

The conducted external validation confirmed statistically significant differences in the levels of four out of six metabolites that were selected as LC marker candidates by means of untargeted metabolomics profiling (Fig. 1b). Patients’ stratification based on smoking status impacted the discriminating ability of the selected metabolite LC marker candidates in a more pronounced way than in the verification step (Supplementary Tables S6 and S7). In a study of serum metabolic profiles of male LC patients in South Korea statistical analyses based on different comparison groups were also performed to find the association of observed metabolic alterations with a smoking habit of the subjects^43^. Another strategy in metabolomics research of LC is to integrate smoking data into any developed diagnostic model^44^.

The applied multi-step metabolomics workflow allowed us to propose some compounds as promising molecules for further testing as LC markers. We observed downregulation in serum levels of allantoin in LC patients compared to COPD patients. Allantoin is the main oxidation product of urate, a potent antioxidant in human body fluids covering up to 65% of the total free radical scavenging capacity of plasma^45,46^. Because humans lack uricase that converts urate to allantoin, the levels of allantoin in blood and urine have been suggested as a biomarker of oxidative stress^47,48^. However, the utility of blood allantoin levels in oxidative stress studies is limited by its low micromolar range requiring sensitive analytical techniques, such as mass spectrometry^49,50^. The elevated levels of allantoin occur in inflammation-related diseases, such as rheumatoid arthritis and coronary artery disease^51–53^. What is interesting, a study of a range of oxidative damage biomarkers demonstrated that different biomarkers reflect different aspects of oxidative stress. Certain markers were increased in smokers even after a period of abstinence from cigarette smoking, whereas another panel of markers (including allantoin) was increased after acute smoking^54^. Cigarette smoke is rich in reactive oxygen species (ROS), which contribute to inflammation, which by further generation of ROS may contribute to cancer initiation and progression^55^. As both LC and COPD are oxidative stress-related diseases, allantoin cannot play the role of a stand-alone biomarker of any of them.

Another molecule that exhibited altered serum concentration in LC patients, in both the discovery set and the validation set, was glutamic acid. Glutamic acid is one of the proteinogenic amino acids involved in plenty of physiological and pathophysiological processes. It is an excitatory neurotransmitter in the central nervous system and conducts signals through binding with different types of receptors. Increasing evidence has shown the involvement of this metabolite or its salt glutamate in cancer development^56^. Glutamate can fuel the TCA cycle intermediates, which results in the release of ATP and provides the required energy for tumor growth. Moreover, glutamate helps maintain redox homeostasis as together with glycine and *l*-cysteine is required for glutathione synthesis^57^. Glutamic acid was among the discriminating metabolites in previous LC metabolomics studies; however, the results of the research showed some discrepancies regarding its blood level in cancer patients. Some studies revealed upregulation of glutamic acid in patients with non-small cell LC relative to healthy controls^58,59^, whereas other studies revealed downregulation of this amino acid in LC patients relative to healthy controls^28^. The current study showed a decreased serum level of glutamic acid in LC patients relative to COPD patients and the type of a comparative group can justify the observed inconsistency. Vanhove et al.^60^ performed NMR-based metabolic profiling of three groups of patients: patients with LC, patients with lung inflammation, and healthy control group. They indicated that cancer patients had lower relative plasma concentration of glutamate compared to patients with inflammation and based on the performed data analysis they proposed measurement of the plasma glutamate concentration as a complementary method to differentiate benign PET-positive lung lesions from lung cancer. Moreover, glutamate was also found a smoking-related metabolite exhibiting higher serum levels in smokers compared to nonsmokers^16,20^. Due to the remarkable metabolic versatility of glutamate, it is difficult to unambiguously interpret its increased or decreased level in LC patients' blood. Nevertheless, the identification of the altered level of this amino acid in cancer patients proves its central role in metabolic processes influencing tumor progression^56^.

The present study underscores the importance of the evaluation of tobacco smoking in metabolomics studies of LC and other respiratory diseases. Adding information on environmental exposure or lifestyle factors to acquired metabolomics data makes their interpretation more reliable. One way to study tobacco-related diseases is to focus not only on biomarkers of effect (i.e., molecules related to organ reactions) but also on biomarkers of exposure (i.e., metabolites of smoke)^20^. Markers such as cotinine, a circulating metabolite of nicotine, may add to a better assessment of the smoking intensity of patients than self-reporting^61^. As experience is gained, it is likely that more well-designed metabolomics studies providing clinically relevant findings will be undertaken in the future. As almost all LC patients possess a smoking background, the incorporation of data on tobacco smoking would benefit the omics findings.

Finally, our study has several strengths and limitations that should be recognized. The strength of the study is the matching of the LC group and the COPD group in terms of smoking habits. Therefore, we minimized any potential bias from unbalanced groups of patients during data analysis. Moreover, we adopted strict inclusion criteria into the group of current smokers and former smokers, which allowed us to explore differences in metabolite profiles related to not only the occurrence of lung cancer but also linked to distinct smoking status. Unlike many LC metabolomics studies, the discovery-based (untargeted) metabolomics stage was followed by the verification stage and validation stage using quantitative methods and a new set of samples. An absence of never-smokers in the study cohorts is one of the study limitations. LC is predominantly linked to cigarette smoking; however, it is estimated that 10–25% of cases have no history of smoking^62^. Thus, a collection of a relatively large sample size for never-smokers takes much more time than a sample size for smokers and past smokers. It should be mentioned that LC in smokers and never-smokers possess different molecular profiles and distinct tumor microenvironments^63^, which makes this topic also very interesting from the metabolomics point of view. A limitation of the untargeted step is the application of only one analytical platform (LC-HRMS). Metabolites exhibit extremely different properties in terms of polarity, pK_a_, pH, solubility, chemical and thermal stability, etc^64^. Therefore, the study of global metabolite profile presents a great analytical challenge, and different analytical platforms can complement one another in providing metabolite coverage of a sample. To acquire a broad metabolite profile of the collected samples, we applied a single extraction-dual separation workflow. However, using two types of chromatographic columns we identified no nicotine metabolites, which could enrich the interpretation of the observed differences in the metabolome between the compared patient groups. Another weakness of the study is the lack of separate statistical analysis for each histological type of LC (adenocarcinoma and squamous-cell carcinoma). The purpose of the study was to explore how the serum metabolome of LC patients differs from the metabolome of the non-cancer smoking status-matched control group. We focused on the influence of tobacco smoking on the acquired metabolite profiles rather than on the influence of cancer histology. Therefore, this issue should be addressed in future investigations.

## Conclusions

The study supports the importance of including data on patient smoking background in metabolomics data analysis since it allows elimination of false positives and a better understanding of the shifts in metabolite profiles in cancer patients. We examined serum metabolite profiles of LC and COPD patients, paying special attention to their smoking status. The performed statistical analyses demonstrated that among metabolites with the highest discriminating potential were allantoin, glutamic acid, and succinic acid. Given the study design, the results are not confounded by different smoking exposures of patients. Due to the diverse roles of the mentioned molecular species in metabolic regulations, their role as LC markers has to be clarified.

## Methods

### Chemicals and reagents

Solvents and reagents employed were of HPLC or LC–MS grade. Acetonitrile, chloroform, isopropanol, methyl tert-butyl ether (MTBE) were purchased from Merck Millipore (Darmstadt, Germany). Methanol and formic acid were purchased from Fisher-Scientific (Loughborough, UK). Ultrapure water (18.2 MΩ/cm resistivity at 25 °C) was from Direct-Q3 UV water purifying system (Merck Millipore, Darmstadt, Germany). Standards of metabolites and internal standards were bought from Sigma Aldrich (St. Louis, MO, USA). The reference mass solution kit and tuning mix for calibrating the Q-TOF–MS were purchased from (Agilent Technologies, Santa Clara, CA, USA).

### Patients, sample collection and study design

The study was performed in accordance with the Declaration of Helsinki^65^ and was approved by the ethics committee at the Poznan University of Medical Sciences (Decision No. 746/17 and 314/18). Written, informed consent was obtained from all study participants before sample collection. Patients who had agreed to participate in the study completed a survey about smoking habit, concomitant diseases and medicines taken in the presence of medical staff. Detailed information about tobacco smoking such as smoking status (current, former, never); smoking intensity; and time since smoking cessation were obtained from all patients included in the study. A total of 155 patients were included in the study: 78 individuals with newly diagnosed LC and 77 patients with COPD that constituted a matched non-cancer group. All cancer patients had histopathologically confirmed non-small cell lung cancer (NSCLC). None of the patients received chemotherapy or radiotherapy and had other cancers. COPD patients without respiratory failure and oxygen therapy, at the moderate stage of the disease were selected for the study. Moreover, a criterion for the absence of malignant disease was met in this group of patients. Patient clinical and demographic characteristics are presented in Table 1.

Blood samples were collected after overnight fasting in the Department of Thoracic Surgery and the Department of Pulmonology, Allergology and Respiratory Oncology, Poznan University of Medical Sciences. For serum isolation, blood was collected into tubes with a clotting activator (S-Monovette system, Sarstedt, Nümbrecht, Germany) according to the manufacturer’s instruction. The sera were aliquoted and stored at − 80 °C until use.

Both, the LC group and COPD group were divided into a discovery set and a validation set (Table 1). The discovery set was used for untargeted metabolic profiling to propose serum metabolome traits of cancer patients, which were then validated in a new set of samples and targeted quantitation methods. Moreover, to better assess the influence of smoking on the studied metabolic profiles, patients were also divided into subgroups according to their smoking status. Only current and former smokers were enrolled in the research. The eligibility criteria for current smokers were: cigarette smoking for at least 6 months of 10 cigarettes per day or more and smoking intensity of at least 10 packyears (i.e. a stable smoking pattern). The eligibility criterion for former smokers was a smoking cessation time of at least two years.

### Untargeted metabolomics

#### Sample preparation

Sera were thawed on ice. Then protein precipitation was conducted by adding 350 µL of ice-cold methanol to 100 µL of serum. The samples were vigorously vortexed for 20 s and centrifuged (13,000*g*, 10 min, 4 °C). An aliquot of the supernatant was transferred to a chromatographic vial and was subjected to liquid chromatography-quadrupole time-of-flight mass spectrometry (LC-Q-TOF–MS) analysis. To avoid possible bias and batch effect, samples were prepared in random order on the same day as one batch. Samples were also randomized for the following LC–MS analyses. Additionally, 50 µL of supernatant from each sample was taken and pooled to obtain a quality control (QC) sample, which was analyzed at regular intervals throughout the sequence.

#### Instrumentation

Liquid chromatography-high resolution mass spectrometry (LC-HRMS) untargeted metabolomics analyses were performed using a 1290 Infinity II UHPLC system coupled to a 6550 iFunnel Q-TOF mass spectrometer equipped with a dual AJS electrospray ionization source (Agilent Technologies, Santa Clara, CA, USA).

Chromatographic separation of polar metabolites was conducted on a SeQuant ZIC-HILIC (Merck Millipore, Darmstadt, Germany) column (100 × 2.1 mm, 3.5 μm particle size) connected to a guard column (2.1 × 2 mm, 3.5 μm particle size) (HILIC separation). The column was maintained at 25 °C. The injection volume was 2 μL and the flow rate was 0.3 mL/min. The gradient elution of solvent A (0.1% formic acid in water) and solvent B (0.1% formic acid in acetonitrile) was programmed as follows: 0–1.5 min with 95% B, 1.5–12 min from 95 to 40% B, 12–14 min with 40% B, 14–14.2 min from 40 to 25% B, 14.2–17 min with 25% B; 17–18 min from 25 to 95% B, 18–25 min with 95% B.

Chromatographic separation of lipid metabolites was conducted on a Zorbax Eclipse Plus C18 (Agilent Technologies, Santa Clara, CA, USA) column (2.1 × 100 mm, 1.8 µm particle size) connected to a guard column (2.1 × 2 mm, 3.5 μm particle size) (RP-LC separation). The column was maintained at 50 °C. The injection volume was 2 μL and the flow rate was 0.4 mL/min. The mobile phase comprised 0.1% formic acid in water (solvent A) and 2-propanol:acetonitrile (90:10, *v*/*v*) with 0.1% formic acid (solvent B). The gradient conditions were as follows: 0–3 min in 5% B, 3–5 min from 5 to 30% B, 5–18.5 min from 30 to 98% B, 18.5–20 min with 98% B, 20–20.5 min from 98 to 5% B, 20.5–25 min with 5% B.

Data were collected in positive and negative ionization mode. The following ion source parameters were applied: drying and sheath gas temperature, 250 °C; drying gas flow, 15 L/min; sheath gas flow, 8 L/min; nebulizer pressure, 35 psig; fragmentor voltage, 380 V; capillary voltage, + 3000 V (positive mode) or − 3000 V (negative mode). The mass spectrometer operated in all ion fragmentation (AIF) acquisition mode with a mass range of 50–1200 *m/z*. In AIF mode, a high-resolution full scan was acquired comprising three experiments at three alternating collision energies (0 eV, 10 eV, 30 eV). The acquisition rate was 6 scans/s. The mass spectrometer was calibrated and tuned according to the manufacturer’s instructions. The details of the applied UHPLC-Q-TOF–MS/MS methodology were described previously^66^.

#### Data processing and quality control

The obtained raw LC-HRMS data files were processed using Profinder version B.06 (Agilent Technologies, Santa Clara, CA, USA) along with personal compound database libraries (PCDLs). Separate PCDLs, depending on the chromatographic column and ionization mode, were applied. The construction of the in-house metabolite databases was described previously^66^. The metabolite identification was based upon accurate mass and retention time (AMRT) confirmation as well as fragment ion identification at two collision energies (MS/MS data). To obtain the broadest coverage of serum metabolome, MS-DIAL version 3.4^67^ was additionally used for deconvolution, peak picking, alignment, and metabolite identification (similarity calculation was based on precursor *m*/*z*, isotopic ratios, and MS/MS spectrum with the reference databases).

For all metabolites identified in the untargeted metabolomics experiment coefficient of variation (%CV) for abundances in QCs (n = 14) was calculated, which allowed for quantitative calculation of method precision. To remove from the datasets not fully reproducible variables only features with %CV value below 15% were kept for further statistical analyses. Additionally, QC samples’ consistency was verified using principal component analysis (PCA). For compounds identified in both ionization modes (positive and negative), the polarity with higher signal intensity and/or lower coefficient of variation (%CV) calculated for QC samples was chosen.

### Targeted metabolomics

#### Sample preparation

For the determination of polar metabolites, an aliquot of serum (100 µL) was mixed with 12 µL of internal standard solution (IS, succinic acid-d_6_, c = 5 mM) and 488 µL of ice-cold methanol. After vortexing (1400 rpm, 3 min) and centrifugation (14,000*g*, 10 min), an aliquot of the supernatant was transferred to a chromatographic vial.

For the determination of lipid metabolites, an aliquot of serum (50 µL) was mixed with 10 µL of IS solution (sphingosine-1-phosphate (d17:1), c = 10 µM). Then extraction step was performed according to the protocol proposed by Pellegrino et al.^68^. One milliliter of methanol: methyl tert-butyl ether (MTBE):chloroform, 1.33:1:1 (*v*/*v*/*v*) mixture was added, then the samples were vortex-mixed (30 s), shook (20 min, 1000 rpm, room temperature) and centrifuged (5 min, 3000 rpm, room temperature). The final step included evaporation in a vacuum concentrator (miVac Duo Concentrator, Genevac, Stone Ridge, NY, USA), reconstitution of a dry residue in 1 mL of methanol (that yielded dilution factor of 20), vortexing and transferring to a chromatographic vial.

#### Instrumentation

The targeted analyses were performed using 1260 Infinity HPLC system (Agilent Technologies, Santa Clara, CA, USA) coupled to a 4000 QTRAP triple quadrupole mass spectrometer equipped with a TurboV electrospray ionization source (Sciex, Framingham, MA, USA).

For determination of polar metabolites (allantoin, glutamic acid, inosine, succinic acid), Kinetex HILIC (Phenomenex, Torrance, CA, USA) column (150 × 3 mm, 2.6 μm particle size) was used. An isocratic elution of 0.2% formic acid in water:0.2% formic acid in methanol (10:90,* v*/*v*) at a flow rate of 0.25 mL/min was applied. The column was maintained at 50 °C and the injection volume was 5 μL. The run time was 6 min. A negative ionization mode was applied. The source parameters were set as follows: curtain gas, 30 psig; temperature, 600 °C; ion source gas 1, 60 psig; ion source gas 2, 60 psig; IonSpray voltage, − 4500 V.

For determination of lipid metabolites (sphingosine-1-phosphate (d18:1), 1–2-dioleoyl-sn-glycerol), XTerra MS C18 (Waters, Milford, MA, USA) column (100 × 2.1 mm, 3.5 μm particle size) was used. The gradient elution of solvent A (0.1% formic acid in water:acetonitrile, 8:2, *v*/*v*) and solvent B (0.1% formic acid in isopropanol:acetonitrile, 8:2, *v*/*v*) was programmed as follows: 0–1 min from 30 to 70% B, 1–2 min from 70 to 95% B, 2–6 min with 95% B, 6–6.5 min from 95 to 30% B; 6.5–12 min with 30% B. Other chromatographic parameters were as follows: flow rate, 0.4 mL/min; column temperature, 60 °C; injection volume, 2 µL. A positive ionization mode was applied. The source parameters were set as follows: curtain gas, 40 psig; temperature, 500 °C; ion source gas 1, 60 psig; ion source gas 2, 60 psig; IonSpray voltage, 5000 V.

The MS/MS analyses were conducted in a multiple reaction monitoring (MRM) mode. For each compound, two MRM transitions were monitored. The transition with the most abundant product ion was selected for quantification, whereas the second transition was used for identity confirmation. The transitions and optimized parameters for each of the determined compounds are listed in Supplementary Table S1. Dwell time was set at 67 ms and 100 ms for polar and lipid metabolites, respectively.

#### Methods’ validation

Targeted LC–MS/MS methods were validated according to the European Medicines Agency (EMA) guidelines on bioanalytical method validation^36^. The methods’ selectivity was achieved by employing MRM mode with two transitions monitored. The discrimination between analytes and potential interferences was obtained by the comparison of retention time, two MRM transitions and MRM ratio with the respective values recorded for the neat standard solution of a given metabolite. This allowed to acquire four identification points for each metabolite, which is recommended by the European Union (EU) Guidelines^69^. However, due to the same mass and similar fragmentation of inosine and allopurinol riboside, a risk of signal contribution occurs. Therefore, separation of these two metabolites should be addressed in the future. The calibration samples were prepared by mixing of standard solutions of analytes, IS solution and 2% bovine albumin solution in PBS used as a surrogate matrix^70^. The calibration curves were constructed using linear regression, and their ranges were adjusted to different concentration levels of analytes observed in a pilot set of serum samples and based on available literature^51,71–74^. The limit of quantification (LOQ) was established as the lowest standard concentration on the calibration curve having acceptable values of accuracy (bias ≤ 20%) and precision (CV ≤ 20%) (Supplementary Table S2). Quality control samples (QC), used for the determination of precision and accuracy of the methods, were prepared by spiking surrogate matrix with known quantities of analytes. During the validation, three concentration levels of QC samples were analyzed for each analyte along with the LOQ sample. To calculate intra-run precision and accuracy, each QC sample was prepared and analyzed five times in a single run. The inter-run precision and accuracy were estimated by analyzing five replicates of each QC sample in three different runs. The high precision (CV < 13.8%) and accuracy (%bias < 14.8%) of targeted methodologies were obtained. The absolute recovery values, obtained based on peak areas of analytes, were in the range from 69.4 to 84.8%. Matrix effects was calculated using peak areas of ISs spiked into protein precipitated serum (n = 6) and reference samples (standard solutions). The obtained matrix effects were < 10%. The stability tests included postpreparative stability (autosampler stability), bench-top stability, and freeze–thaw stability. Analytes’ concentrations did not change more than ± 15% after 24 h storage of samples and calibrators in autosampler at 4 °C in comparison to the values obtained directly after preparation. Bench-top stability was assessed by keeping three serum samples at ambient temperature for 5 h before the sample preparation procedure, whereas freeze–thaw stability was assessed by thawing samples three times before the sample preparation procedure. Both tests indicated a significant decrease (> 15%) of inosine concentration. The selected validation parameters of the targeted LC–MS/MS methods are presented in Supplementary Table S2.

### Data analysis

**Figure 2:**
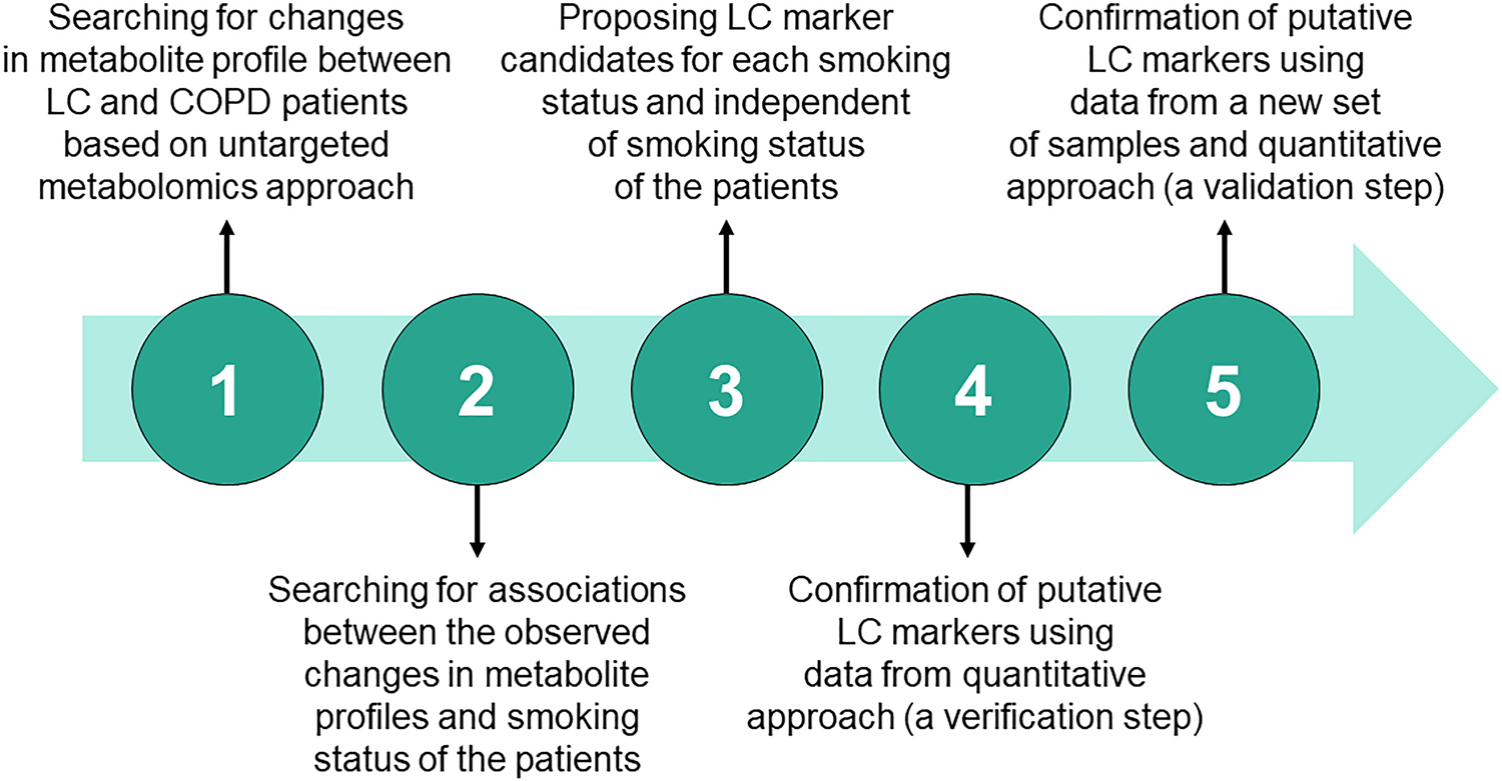
Workflow of the performed data analysis. *LC* lung cancer, *COPD* chronic obstructive pulmonary disease.

The performed statistical analysis consisted of several steps and aimed not only to detect differences between LC and COPD groups but also to detect metabolites associated with the smoking status of the patients (Fig. 2). For data analysis MetaboAnalyst 4.0^75^, TIBCO Statistica 13.3, Graphpad Prism 8 were employed. In the untargeted metabolomics dataset analysis, only metabolites consistently detected in samples were used for statistical tests. In the first step of statistical data analysis, sample grouping was checked using principal component analysis (PCA). Before PCA, autoscaling (scaling to unit variance) was applied. Significant differences between sample groups and subgroups were detected using volcano plots, which combine the size of the fold change and the statistical significance level (a *P* value for a t-test of differences between samples). The volcano plot was created using fold change threshold of 1.3 and an FDR-adjusted *P* value (calculated using Benjamini and Hochberg method) threshold of 0.05. Analysis of data acquired in a targeted quantitative approach was conducted using univariate statistical tests. Analysis of data acquired in a targeted quantitative approach was conducted using univariate statistical tests. In the first step, the normality of the data distribution of each variable was checked using the Shapiro–Wilk test. The Mann–Whitney U test was applied to compare the concentrations of metabolites that did not have a normal distribution. For variables exhibiting a normal distribution, Levene’s test was used to examine the equality of variances. To detect the significant differences in metabolite concentrations between the groups, the Student’s t-test was used for variables with equal variances, and the Welch’s F test was applied for variables with unequal variances. In all tests, a P value below 0.05 was considered statistically significant.

### Supplementary Information

Supplementary Information.

## Data Availability

Data is provided within the manuscript or supplementary information files.
