# Supplementary material for: Metabolomics-based search for lung cancer markers among patients with different smoking status
Source: Sci Rep. 2024 Jul 4;14:15444. doi: 10.1038/s41598-024-65835-2 (PMC11224321; doi:10.1038/s41598-024-65835-2)
Supplement: Supplementary file 1 — Supplementary Information. [file 41598_2024_65835_MOESM1_ESM.pdf]

SUPPLEMENTARY INFORMATION

Metabolomics-based search for lung cancer markers among patients with different smoking status

Agnieszka Klupczynska-Gabryszak[1*], Evangelia Daskalaki[2], Craig E. Wheelock[2,3], Mariusz Kasprzyk[4], Wojciech Dyszkiewicz[4], Marcin Grabicki[5], Beata Brajer-Luftmann[5], Magdalena Pawlak[1], Zenon J. Kokot[6], Jan Matysiak[1]

[1] Department of Inorganic and Analytical Chemistry, Poznan University of Medical Sciences, Poznan, Poland
[2] Unit of Integrative Metabolomics, Institute of Environmental Medicine, Karolinska Institutet, Stockholm, Sweden
[3] Department of Respiratory Medicine and Allergy, Karolinska University Hospital, Stockholm, Sweden
[4] Department of Thoracic Surgery, Poznan University of Medical Sciences, Poznan, Poland
[5] Department of Pulmonology, Allergology and Respiratory Oncology, Poznan University of Medical Sciences, Poznan, Poland
[6] Faculty of Health Sciences, Calisia University, Kalisz, Poland
* corresponding author: aklupczynska@ump.edu.pl

Contents

Supplementary Table S1. Compound-dependent parameters for analytes and internal standards in MRM mode for targeted LC-MS/MS analysis.

Supplementary Table S2. Validation parameters of the targeted LC-MS/MS methods.

Supplementary Table S3. Results of univariate statistical analysis of metabolites determined in targeted quantitative analysis in the discovery set of samples. Bold type for P values indicates statistical significance.

Supplementary Table S4. Results of univariate statistical analysis of metabolites determined in targeted quantitative analysis in the validation set of samples. Bold type for *P* values indicates statistical significance ($P < 0.05$). LC: lung cancer; COPD: chronic obstructive pulmonary disease.

Supplementary Table S5. Metabolites identified in the studied serum samples using LC-HRMS-based untargeted methodology (arranged alphabetically).

Supplementary Table S6. Serum concentration levels of the six lung cancer candidate markers determined using targeted metabolomics analysis among current smokers in the discovery set and the validation set of samples. LC: lung cancer; COPD: chronic obstructive pulmonary disease.

Supplementary Table S7. Serum concentration levels of the six lung cancer candidate markers determined using targeted metabolomics analysis among past smokers in the discovery set and the validation set of samples. LC: lung cancer; COPD: chronic obstructive pulmonary disease.

Supplementary Figure S1. Scores plot between the first and the second principal components (with the explained variances shown in brackets) obtained in the principal component analysis of HILIC-based untargeted metabolite profiles (A and B) and RP-LC-based untargeted metabolite profiles (C and D). COPD: samples from patients with chronic obstructive pulmonary disease; LC: samples from patients with lung cancer; QC: quality control samples.

Supplementary Table S1. Compound-dependent parameters for analytes and internal standards in MRM mode for targeted LC-MS/MS analysis.

Compound	Retention time (min)	Molecular weight (amu)	Polarity	Precursor ion	MRM transition	DP (V)	EP (V)	CE (V)	CXP (V)
**Analytes**									
allantoin	2.80	158.12	neg	$[M-H]^-$	T1: 157.0 → 42.0	-70	-10	-35	-5
					T2: 157.0 → 113.9	-70	-10	-16	-16
glutamic acid	2.97	147.13	neg	$[M-H]^-$	T1: 145.9 → 102.0	-40	-10	-19	-10
					T2: 145.9 → 127.9	-40	-10	-15	-10
inosine	2.81	268.23	neg	$[M-H]^-$	T1: 266.9 → 134.9	-85	-9	-30	-10
					T2: 266.9 → 91.9	-85	-9	-55	-15
succinic acid	2.65	118.09	neg	$[M-H]^-$	T1: 117.0 → 99.0	-45	-9	-16	-16
					T2: 117.0 → 73.0	-45	-9	-18	-12
1-2-dioleoylglycerol	5.49	620.99	pos	$[M+NH_4]^+$	T1:.638.6 → 603.6	80	5	25	30
					T2: 638.6 → 339.5	80	5	35	20
sphingosine-1-phosphate (d18:1)	4.10	379.47	pos	$[M+H]^+$	T1: 380.3 →264.30	53	5	25	18
					T2: 380.3 → 247.3	53	5	20	18
**Internal standards**									
succinic acid-d_6	2.65	124.12	neg	$[M-H]^-$	T2: 123.0 → 79.0	-19	-9	-16	-12
					T1: 123.0 → 77.0	-19	-9	-16	-14
sphingosine-1-phosphate (d17:1)	3.88	365.45	pos	$[M+H]^+$	T1: 366.3 → 250.3	75	5	25	15
					T2: 366.3 → 233.3	75	5	16	13

T1: quantification transition; T2: confirmation transition; DP: declustering potential; EP: entrance potential; CE: collision energy; CXP: collision cell exit potential

Supplementary Table S2. Validation parameters of the targeted LC-MS/MS methods.

Analyte	Calibration range	Correlation coefficient, r	QC concentration	Accuracy (%)		Precision (%CV)	
				Intra-day	Inter-day	Intra-day	Inter-day
allantoin	0.1-20 μM	0.993	0.1 μM (LOQ)	116.40	119.60	11.23	12.83
			1.5 μM	112.13	113.15	6.27	12.27
			7.5 μM	111.44	111.23	3.76	3.96
			16 μM	109.06	108.92	3.67	4.19
glutamic acid	5-200 μM	0.999	5 μM (LOQ)	109.71	109.62	7.59	7.88
			15 μM	99.67	105.37	6.33	12.36
			100 μM	103.83	102.23	5.50	6.32
			160 μM	99.44	102.66	4.30	5.23
inosine	0.5-20 μM	0.998	0.5 μM (LOQ)	115.28	108.99	7.15	9.06
			1.5 μM	104.92	106.7	6.76	7.31
			7.5 μM	114.83	111.63	2.69	8.5
			16 μM	111.41	109.6	4.6	6.82
succinic acid	0.5-20 μM	0.999	0.5 μM (LOQ)	94.58	93.12	17.43	18.39
			1.5 μM	95.07	95.14	12.93	13.84
			7.5 μM	107.12	102.72	3.77	7.25
			16 μM	103.44	102.14	4.47	6.49
1-2-dioleoylglycerol	50–1500 nM	0.996	50 nM (LOQ)	119.00	114.50	16.80	15.37
			150 nM	92.83	96.33	2.71	7.16
			600 nM	89.58	97.11	3.19	12.80
			1200 nM	103.33	105.14	3.67	4.24
sphingosine-1-phosphate (d18:1)	5–500 nM	0.997	5 nM (LOQ)	120.65	120.77	1.76	3.38
			15 nM	91.33	94.76	4.17	6.21
			125 nM	96.40	93.71	1.59	4.05
			400 nM	98.19	96.82	2.09	2.78

LOQ: limit of quantification; QC: quality control sample; RSD: relative standard deviation

Supplementary Table S3. Results of univariate statistical analysis of metabolites determined in targeted quantitative analysis in the discovery set of samples. Bold type for *P* values indicates statistical significance.

Comparison type	LC vs COPD				
Compound	Shapiro-Wilk test		Mann-Whitney U test	Levene's test	Student's t-test
	P value for LC group	*P* value for COPD group	*P* value	*P* value	*P* value
allantoin	**0.000032**	**<0.000001**	**<0.000001**		
glutamic acid	**0.000123**	0.888274	**<0.000001**		
inosine	**0.027264**	**<0.000001**	**<0.000001**		
succinic acid	**0.000056**	**<0.000001**	**<0.000001**		
sphingosine-1-phosphate (d18:1)	0.245278	0.561716		0.492249	**0.000004**
1-2-dioleoylglycerol	**0.000001**	**0.000055**	**0.001046**		
Comparison type	LC smokers vs COPD smokers				
Compound	Shapiro-Wilk test		Mann-Whitney U test	Levene's test	Student's t-test
	P value for LC smokers	*P* value for COPD smokers	*P* value	*P* value	*P* value
allantoin	0.176929	**0.000001**	**0.000001**		
glutamic acid	**0.016122**	0.525534	**0.000018**		
inosine	**0.033327**	**0.000043**	**0.003499**		
succinic acid	**0.005186**	**0.000007**	**0.000009**		
sphingosine-1-phosphate (d18:1)	0.257119	0.957847		0.201051	**0.000176**
1-2-dioleoylglycerol	**0.000062**	**0.007754**	**0.043603**		
Comparison type	LC past smokers vs COPD past smokers				
Compound	Shapiro-Wilk test		Mann-Whitney U test	Levene's test	Student's t-test
	P value for LC past smokers	*P* value for COPD past smokers	*P* value	*P* value	*P* value
allantoin	**0.001676**	**0.000197**	**0.000013**		
glutamic acid	**0.007476**	0.231163	**0.000479**		
inosine	0.329283	**<0.000001**	**0.000007**		
succinic acid	**0.027477**	**0.000901**	**0.000122**		
sphingosine-1-phosphate (d18:1)	0.243349	0.347993		0.966539	**0.004012**
1-2-dioleoylglycerol	**0.000345**	**0.000175**	**0.002635**		

Supplementary Table S4. Results of univariate statistical analysis of metabolites determined in targeted quantitative analysis in the validation set of samples. Bold type for *P* values indicates statistical significance (*P* < 0.05). LC: lung cancer; COPD: chronic obstructive pulmonary disease.

Comparison type	LC vs COPD					
Compound	Shapiro-Wilk test		Mann-Whitney U test	Levene's test	Welch's F test	Student's t-test
	P value for LC group	*P* value for COPD group	*P* value	*P* value	*P* value	*P* value
allantoin	**0.000801**	0.101553	**0.000085**			
glutamic acid	**0.000248**	**0.023542**	**0.000119**			
inosine	**0.000436**	**0.000259**	0.266497			
succinic acid	**0.000785**	**0.001520**	**0.003305**			
sphingosine-1-phosphate (d18:1)	0.399357	0.261486		0.556797		**0.007363**
1-2-dioleoylglycerol	**0.038872**	0.085327	0.495332			
Comparison type	LC smokers vs COPD smokers					
Compound	Shapiro-Wilk test		Mann-Whitney U test	Levene's test	Welch's F test	Student's t-test
	P value for LC smokers	*P* value for COPD smokers	*P* value	*P* value	*P* value	*P* value
allantoin	**0.008630**	0.490249	**0.014299**			
glutamic acid	**0.041140**	0.085687	**0.001099**			
inosine	**0.041132**	0.224278	0.438212			
succinic acid	**0.000060**	0.194008	**0.001130**			
sphingosine-1-phosphate (d18:1)	0.663676	0.696158		0.731295		0.050331
1-2-dioleoylglycerol	0.056663	0.388644		0.606437		0.940750
Comparison type	LC past smokers vs COPD past smokers					
Compound	Shapiro-Wilk test		Mann-Whitney U test	Levene's test	Welch's F test	Student's t-test
	P value for LC past smokers	*P* value for COPD past smokers	*P* value	*P* value	*P* value	*P* value
allantoin	0.853408	0.280722		**0.000668**	**0.000502**	
glutamic acid	**0.035887**	0.272297	**0.041432**			
inosine	**0.003177**	**0.000339**	0.597029			
succinic acid	0.667040	0.545120		0.260160		0.546714
sphingosine-1-phosphate (d18:1)	0.903636	0.861571		0.189505		0.067842
1-2-dioleoylglycerol	0.310208	**0.043282**	0.290350			

Supplementary Table S5. Metabolites identified in the studied serum samples using LC-HRMS-based untargeted methodology (arranged alphabetically).

No	**Compound Name**	**Formula**	**Molecular weight**	**HMDB ID**	**Separation mode**
1	4-Hydroxyproline	C5H9NO3	131.05824	HMDB0000725	HILIC
2	Aconitic acid	C6H6O6	174.01644	HMDB0000072	HILIC
3	Allantoin	C4H6N4O3	158.04399	HMDB0000462	HILIC
4	Arginine	C6H14N4O2	174.11168	HMDB0000517	HILIC
5	Asparagine	C4H8N2O3	132.05349	HMDB0000168	HILIC
6	Aspartic acid	C4H7NO4	133.03751	HMDB0000191	HILIC
7	Catechol	C6H6O2	110.03678	HMDB0000957	HILIC
8	Citraconic acid	C5H6O4	130.02661	HMDB0000634	HILIC
9	Citric acid	C6H8O7	192.02700	HMDB0000094	HILIC
10	Citrulline	C6H13N3O3	175.09569	HMDB0000904	HILIC
11	Creatine	C4H9N3O2	131.06948	HMDB0000064	HILIC
12	Dimethylarginine	C8H18N4O2	202.14298	HMDB0003334 / HMDB0001539	HILIC
13	Fucose	C6H12O5	164.06847	HMDB0000174	HILIC
14	Glucuronic acid	C6H10O7	194.04265	HMDB0000127	HILIC
15	Glutamic acid	C5H9NO4	147.05316	HMDB0000148	HILIC
16	Glutamine	C5H10N2O3	146.06914	HMDB0000641	HILIC
17	Glyceraldehyde	C3H6O3	90.03169	HMDB0001051	HILIC
18	Guanosine	C10H13N5O5	283.09167	HMDB0000133	HILIC
19	Histamine	C5H9N3	111.07965	HMDB0000870	HILIC
20	Histidine	C6H9N3O2	155.06948	HMDB0000177	HILIC
21	Hydroxybutanoic acid	C4H8O3	104.04734	HMDB0000008	HILIC
22	Hypotaurine	C2H7NO2S	109.01975	HMDB0000965	HILIC
23	Hypoxanthine	C5H4N4O	136.03851	HMDB0000157	HILIC
24	Indoxyl sulfate	C8H7NO4S	213.00958	HMDB0000682	HILIC
25	Inosine	C10H12N4O5	268.08077	HMDB0000195	HILIC
26	Kynurenine	C10H12N2O3	208.08479	HMDB0000684	HILIC
27	Leucine	C6H13NO2	131.09463	HMDB0000687	HILIC
28	Lysine	C6H14N2O2	146.10553	HMDB0000182	HILIC
29	Malic acid	C4H6O5	134.02152	HMDB0000156	HILIC
30	Maltose / Sucrose	C12H22O11	342.11621	HMDB0000163 / HMDB0000258	HILIC
31	Methionine	C5H11NO2S	149.05105	HMDB0000696	HILIC
32	Methylhistidine	C7H11N3O2	169.08513	HMDB0000479	HILIC
33	N-Acetylaspartic acid	C6H9NO5	175.04807	HMDB0000812	HILIC
34	Phenylacetylglutamine	C13H16N2O4	264.11101	HMDB0006344	HILIC
35	Phenylalanine	C9H11NO2	165.07898	HMDB0000159	HILIC
36	Phosphatidylethanolamine	C41H82NO8P	747.57781	HMDB0060501	HILIC
37	Proline	C5H9NO2	115.06333	HMDB0000162	HILIC
38	Serine	C3H7NO3	105.04259	HMDB0000187	HILIC
39	Succinic acid	C4H6O4	118.02661	HMDB0000254	HILIC
40	Taurine	C2H7NO3S	125.01466	HMDB0000251	HILIC
41	Threonine	C4H9NO3	119.05824	HMDB0000167	HILIC

42	Tryptophan	C11H12N2O2	204.08988	HMDB0000929	HILIC
43	Tyrosine	C9H11NO3	181.07389	HMDB0000158	HILIC
44	Uracil	C4H4N2O2	112.02728	HMDB0000300	HILIC
45	Uric acid	C5H4N4O3	168.02834	HMDB0000289	HILIC
46	Uridine	C9H12N2O6	244.06954	HMDB0000296	HILIC
47	Urocanic acid	C6H6N2O2	138.04293	HMDB0000301	HILIC
48	Valine	C5H11NO2	117.07898	HMDB0000883	HILIC
49	Xanthine	C5H4N4O2	152.03343	HMDB0000292	HILIC
50	1,2-dioleoylglycerol	C39H72O5	620.53798	HMDB0007218	RP-LC
51	1-arachidonoylglycerol	C23H38O4	378.27701	HMDB0011578	RP-LC
52	1-linoleoylglycerol	C21H38O4	354.27701	HMDB0011568	RP-LC
53	2-O-ethyl PAF C-16	C26H56NO6P	509.38453		RP-LC
54	Adrenic acid	C22H36O2	332.27153	HMDB0002226	RP-LC
55	Arachidonic acid	C20H32O2	304.24023	HMDB0001043	RP-LC
56	Arachidonoylcarnitine	C27H45NO4	447.33486	HMDB0006455	RP-LC
57	Ceramide (d18:0/16:0)	C34H69NO3	539.52775	HMDB0011760	RP-LC
58	Ceramide (d18:1/14:0)	C32H63NO3	509.48079	HMDB0011773	RP-LC
59	Ceramide (d18:1/16:0)	C34H67NO3	537.51210	HMDB0004949	RP-LC
60	Ceramide (d18:1/18:0)	C36H71NO3	565.54340	HMDB0004950	RP-LC
61	Ceramide (d18:1/18:1)	C36H69NO3	563.52775	HMDB0004948 / HMDB0011774	RP-LC
62	Ceramide (d18:1/20:0)	C38H75NO3	593.57470	HMDB0004951	RP-LC
63	Ceramide (d18:1/22:0)	C40H79NO3	621.60600	HMDB0004952	RP-LC
64	Ceramide (d18:1/24:0)	C42H83NO3	649.63730	HMDB0004956	RP-LC
65	Ceramide (d18:1/24:1)	C42H81NO3	647.62165	HMDB0004953	RP-LC
66	Chenodeoxycholic acid	C24H40O4	392.29266	HMDB0000518	RP-LC
67	Deoxycholic acid	C24H40O4	392.29266	HMDB0000626	RP-LC
68	Dihomolinoleic acid	C20H34O2	306.25588	HMDB0002925 / HMDB0010378	RP-LC
69	Dihomo-γ-linolenoyl PAF C-16	C44H84NO7P	769.59854		RP-LC
70	Docosahexaenoic acid	C22H32O2	328.24023	HMDB0002183	RP-LC
71	Eicosapentaenoic Acid	C20H30O2	302.22458	HMDB0001999	RP-LC
72	Elaidic Acid	C18H34O2	282.25588	HMDB0000573	RP-LC
73	Glucosylceramide (d18:1/16:0)	C40H77NO8	699.56492	HMDB0004971	RP-LC
74	Glucosylceramide (d18:1/24:1)	C48H91NO8	809.67447	HMDB0004975	RP-LC
75	LacCer(d18:1/16:0)	C46H87NO13	861.61774	HMDB0006750	RP-LC
76	LacCer(d18:1/24:1)	C54H101NO13	971.72729	HMDB0004872	RP-LC
77	Linolenic acid	C18H30O2	278.22458	HMDB0001388 / HMDB0003073	RP-LC
78	Lyso-PAF C-16	C24H52NO6P	481.35322		RP-LC
79	LysoPC(16:0)	C24H50NO7P	495.33249	HMDB0010382	RP-LC
80	LysoPC(18:0)	C26H54NO7P	523.36379	HMDB0010384	RP-LC
81	Myristic acid	C14H28O2	228.20893	HMDB0000806	RP-LC
82	Oleamide	C18H35NO	281.27187	HMDB0002117	RP-LC
83	PAF C-16	C26H54NO7P	523.36379		RP-LC
84	PAF C-18	C28H58NO7P	551.39509		RP-LC

85	Palmitoylcarnitine	C23H45NO4	399.33486	HMDB0000222	RP-LC
86	Palmitoylethanolamide	C18H37NO2	299.28243	HMDB0002100	RP-LC
87	PC(14:0/14:0)	C36H72NO8P	677.49955	HMDB0007866	RP-LC
88	PC(16:0/18:1)	C42H82NO8P	759.57781	HMDB0007972	RP-LC
89	PC(18:0/14:0)	C40H80NO8P	733.56216	HMDB0008031	RP-LC
90	PC(18:1/18:1)	C44H84NO8P	785.59346		RP-LC
91	PE(16:0/18:2)	C39H74NO8P	715.51520	HMDB0008928	RP-LC
92	PE(18:1/16:0)	C39H76NO8P	717.53086	HMDB0009055	RP-LC
93	PE(18:1/18:1)	C41H78NO8P	743.54651	HMDB0009059	RP-LC
94	PG(16:0/18:1)	C40H77O10P	748.52544	HMDB0010574	RP-LC
95	SM(d18:1/12:0)	C35H71N2O6P	646.50497	HMDB0012096	RP-LC
96	SM(d18:1/16:0)	C39H79N2O6P	702.56757	HMDB0010169	RP-LC
97	SM(d18:1/18:0)	C41H83N2O6P	730.59887	HMDB0001348	RP-LC
98	SM(d18:1/18:1)	C41H81N2O6P	728.58322	HMDB0012101 / HMDB0012100	RP-LC
99	SM(d18:1/24:0)	C47H96N2O6P	815.70060	HMDB0011697	RP-LC
100	Sphinganine-phosphate (d18:0)	C18H40NO5P	381.26441	HMDB0001383	RP-LC
101	Sphingosine-1-phosphate (d18:1)	C18H38NO5P	379.24876	HMDB0000277	RP-LC
102	Stearoylethanolamide	C20H41NO2	327.31373	HMDB0013078	RP-LC
103	Taurodeoxycholic acid	C26H45NO6S	499.29676	HMDB0000896	RP-LC

Supplementary Table S6. Serum concentration levels of the six lung cancer candidate markers determined using targeted metabolomics analysis among current smokers in the discovery set and the validation set of samples. LC: lung cancer; COPD: chronic obstructive pulmonary disease.

Metabolite	Verification step					Validation step				
	LC smokers (n=25)		COPD smokers (n=25)		*P* value	LC smokers (n=13)		COPD smokers (n=10)		*P* value
	Mean [μM]	Median [μM]	Mean [μM]	Median [μM]		Mean [μM]	Median [μM]	Mean [μM]	Median [μM]	
allantoin	1.56	1.58	3.13	2.59	0.000001	2.367	2.04	3.51	3.41	0.014299
glutamic acid	92.58	84.30	150.7	160.0	0.000018	91.28	99.80	166.6	146.3	0.001099
inosine	6.05	5.29	2.58	1.49	0.003499	7.347	4.945	4.24	3.90	0.438212
succinic acid	2.87	2.74	4.65	3.76	0.000009	4.93	4.26	7.94	7.68	0.001130
1-2-dioleoylglycerol	11.46	10.78	7.47	7.12	0.043603	16.70	11.38	17.05	17.17	0.940750
sphingosine-1-phosphate (d18:1)	0.74	0.68	0.99	1.04	0.000176	0.80	0.764	1.03	0.99	0.050331

Supplementary Table S7. Serum concentration levels of the six lung cancer candidate markers determined using targeted metabolomics analysis among past smokers in the discovery set and the validation set of samples. LC: lung cancer; COPD: chronic obstructive pulmonary disease.

Metabolite	Verification step					Validation step				
	LC past smokers (n=25)		COPD past smokers (n=25)		*P* value	LC past smokers (n=15)		COPD past smokers (n=17)		*P* value
	Mean [μM]	Median [μM]	Mean [μM]	Median [μM]		Mean [μM]	Median [μM]	Mean [μM]	Median [μM]	
allantoin	1.9734	1.775	3.8468	3.095	0.000013	2.0999	2.1	4.2191	4.54	0.000502
glutamic acid	107.368	105	156.102	145.5	0.000479	124.76	113.5	166.85	161	0.041432
inosine	5.7048	6.02	2.1379	0.805	0.000007	3.9724	1.325	3.2192	2.835	0.597029
succinic acid	3.28	3.00	5.2304	4.485	0.000122	5.0617	4.775	5.3594	5.195	0.546714
1-2-dioleoylglycerol	10.1092	7.60	5.67592	3.28	0.004012	20.06	19.36	15.1094	12.74	0.290350
sphingosine-1-phosphate (d18:1)	0.67236	0.678	0.85256	0.802	0.002635	0.8087	0.768	0.9396	0.944	0.067842

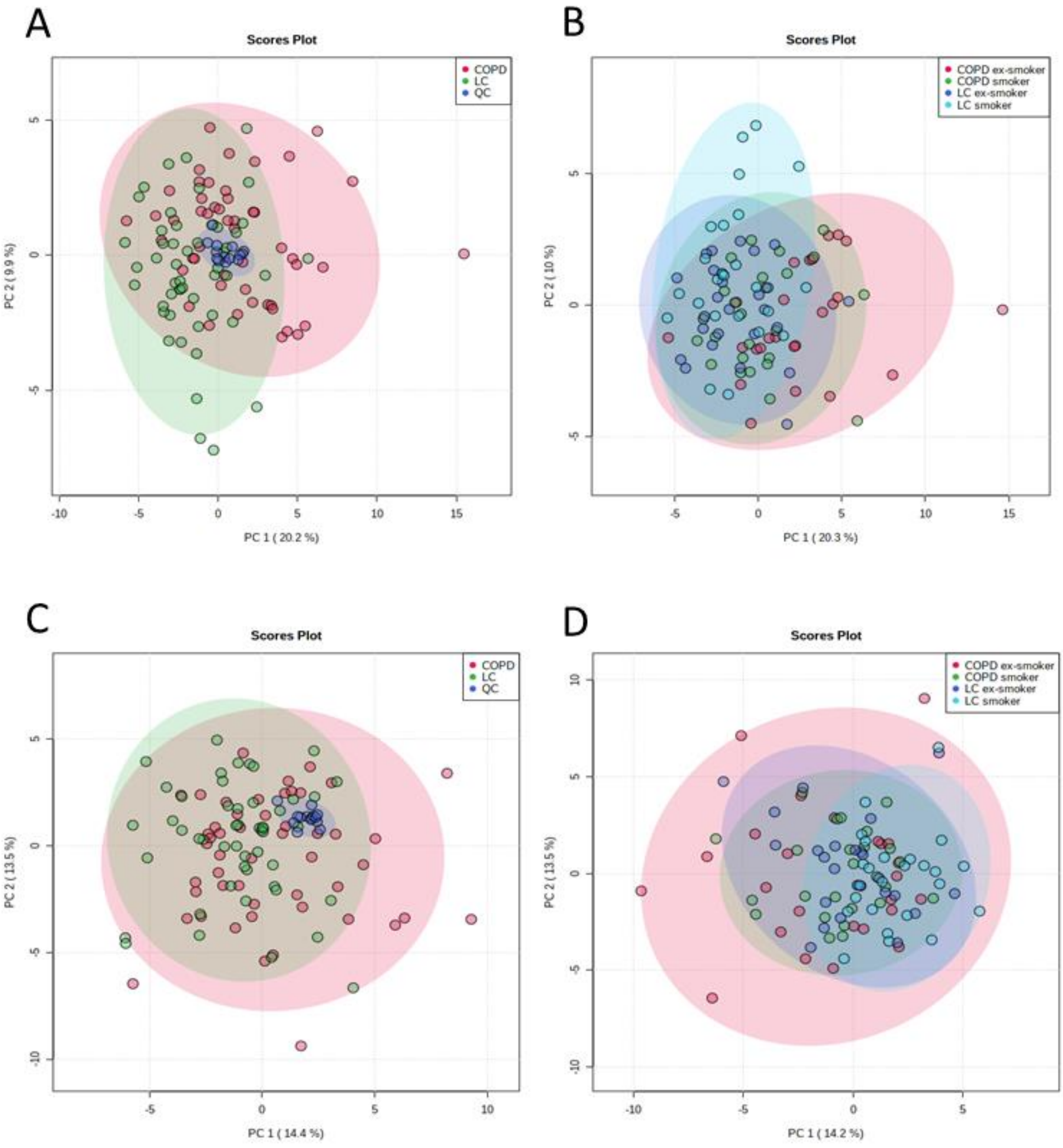

Supplementary Figure S1. Scores plot between the first and the second principal components (with the explained variances shown in brackets) obtained in the principal component analysis of HILIC-based untargeted metabolite profiles (A and B) and RP-LC-based untargeted metabolite profiles (C and D). COPD: samples from patients with chronic obstructive pulmonary disease; LC: samples from patients with lung cancer; QC: quality control samples.
